# The serotonin transporter gene polymorphism (5-HTTLPR) and irritable bowel syndrome: a meta-analysis of 25 studies

**DOI:** 10.1186/1471-230X-14-23

**Published:** 2014-02-10

**Authors:** Zhi-Feng Zhang, Zhi-Jun Duan, Li-Xia Wang, Dong Yang, Gang Zhao, Lin Zhang

**Affiliations:** 1 Department of Gastroenterology, The First Affiliated Hospital of Dalian Medical University, 116000 Dalian, Liaoning province, China; 2 Department of Respiratory Diseases, The First Affiliated Hospital of Dalian Medical University, 116000 Dalian, Liaoning province, China

## Abstract

**Background:**

The results of previous studies assessing the association between the 5-HTTLPR polymorphism of serotonin transporter gene and irritable bowel syndrome (IBS) are inconsistent. The aim of this study was to clarify the association between the 5-HTTLPR mutation and the presence of IBS and its subtypes with a meta-analysis of 25 studies.

**Methods:**

A thorough search for case–control studies evaluating the association between the 5-HTTLPR polymorphism of serotonin transporter gene and the presence of IBS was carried out in four electronic databases. A meta-analysis was performed in accordance with the Cochrane Handbook for systemic reviews.

**Results:**

A total of 25 articles with 3443 IBS cases and 3359 controls were included into our meta-analysis. No significant association was found between this polymorphism and IBS in all populations. Whereas the LL genotype was demonstrated to be a risk factor for constipation predominant IBS (IBS-C) development in the overall population (LL vs SS: OR = 1.570, 95% CI = 1.147-2.148, P = 0.005, Bon = 0.030; LL vs LS: OR = 1.658, 95% CI = 1.180-2.331, P = 0.004, Bon = 0.024; LL vs LS/SS: OR = 1.545, 95% CI = 1.187-2.012, P = 0.001, Bon = 0.006). In the analysis of different ethnicities, L allele and LL genotype were significantly associated with increased IBS-C risk in the East Asian population (L vs S: OR = 1.487, 95% CI = 1.139-1.941, P = 0.003, Bon = 0.018; LL vs SS: OR = 2.575, 95% CI = 1.741-3.808, P = 0.000, Bon = 0.000; LL vs LS: OR = 3.084, 95% CI = 2.017-4.715, P = 0.000, Bon = 0.000; LL vs LS/SS: OR = 2.759, 95% CI = 1.933-3.938, P = 0.000, Bon = 0.000), but not in the Caucasian population.

**Conclusions:**

Different from the conclusions of the earlier meta-analyses, the 5-HTTLPR mutation affects IBS-C but not IBS-D and IBS-M development and this effect only exists in the East Asian population but not other populations.

## Background

Irritable bowel syndrome (IBS) mainly affecting lower gastrointestinal (GI) tracts is a chronic functional GI disorder with an obvious heterogeneity among affected patients. The characteristic presentation of IBS is recurrent abdominal pain accompanied with altered bowel habits [1,2]. According to the Rome III criteria, IBS is categorized into diarrhea predominant IBS (IBS-D), constipation predominant IBS (IBS-C), mixture of diarrhea and constipation IBS (IBS-M) and un-subtyped IBS [3]. IBS is a common disorder encountered by gastroenterologists and its prevalence is approximately 10% [4]. IBS can impair the quality of life and the work efficiency of affected patients. Due to the elusive etiology of IBS, there is still no curative therapy for this condition. Therapy for relieving symptoms is still the mainstay for IBS patients. Long term symptomatic treatment results in an economic burden on not only patients and their families but also healthcare systems [5]. Despite lacking of the definite etiology, some advancements have been achieved in understanding the pathophysiology underlying IBS development in recent years. Gut motility dysfunction, visceral hypersensitivity and psychopathological factors have been implicated to play key roles in the development of IBS [1,3].

Serotonin is a neurotransmitter existing in both central nervous system (CNS) and GI tracts, and regulates GI tracts motility, visceral sensation and mucosal secretion through a paracrine signaling pathway [6]. Previous studies have shown that elevated plasma serotonin is associated with IBS-D and decreased plasma serotonin is associated with IBS-C [7]. Once serotonin is secreted from enterochromaffin (EC) cells, serotonin reuptake transporter (SERT) will be activated to reuptake serotonin back into EC cells and attenuate the effect of serotonin in GI tracts subsequently [8]. Balance between these two opposite processes determining the net tone of serotonin in GI tracts is critical to the maintenance of normal gut functions, especially of GI tracts motility. Changes of the SERT activity would break this balance and could be involved in the development of IBS theoretically. A polymorphism of SERT gene (5-HTTLPR) with a short (S) variation of 14 repeats and a long (L) variation of 16 repeats has been proven to influence the activity of SERT [9-11]. So 5-HTTLPR is very likely to be associated with the development of IBS. Case–control studies about 5-HTTLPR were conducted to verify this hypothesis. Some studies demonstrated a positive association between this polymorphism and IBS [10,11], while another study failed to confirm this association [12]. A meta-analysis of 8 studies conducted in 2007 tried to reach a definite conclusion and showed a negative result [13]. However, the small sample size of this meta-analysis weakened its strength of evidence and this meta-analysis did not terminate the controversy about 5-HTTLPR in IBS development. Another meta-analysis published in a letter showed a positive association between this polymorphism and IBS-C [14]. However, this meta-analysis did not include all the published articles and did not assess the association in different ethnic groups. The third one included most of the publish articles and concluded that 5-HTTLPR was associated with IBS [15]. The third meta-analysis also had limitations and did not assess the association in different IBS subtypes. The association may be different in different IBS subtypes and different ethnic groups. Hence we performed this meta-analysis including all published studies accompanied with ethnic subgroup analyses and IBS subtype analyses to clarify whether 5-HTTLPR was associated with the development of IBS and its subtypes.

## Methods

### Searching strategies

Case–control studies evaluating the association between the 5-HTTLPR polymorphism and IBS were searched in PubMed, Embase and Web of Science with the combinations of the following searching terms: “irritable bowel syndrome”, “IBS”, “serotonin”, “5-Hydroxytryptamine”, “5-HT”, “polymorphism”, “polymorphisms”, “single nucleotide”, “allele” and “genotype”. We also searched China National Knowledge Infrastructure (CNKI) for additional relevant researches. Reference lists of each article, relevant meta-analyses and reviews were searched as well. The last searching date was July 12, 2013.

### Study selection criteria

Studies included in this meta-analysis fulfilled the following selection criteria: 1) Case–control studies with a healthy control arm. 2) Studies evaluating the association between the 5-HTTLPR polymorphism and IBS. 3) IBS diagnosis according to Rome I or II or III criteria. 4) Articles providing allele and genotype frequencies or odds ratios (OR) and 95% confidence intervals (95% CI). Exclusion criteria were as the follows: 1) Republication. 2) Family based studies. If the data of a study was published in different articles, the article with the largest sample size would be included in this meta-analysis. If the data of a study was published not only as an abstract but also as a full text article in different magazines, we selected the full text article to analyze in order to assess the quality of the research more comprehensively. During study selection, two authors would read the highly relevant articles independently to determine eligible studies. A third author would be consulted and the decision would be reached through discussions when a disagreement was encountered.

### Data abstraction

A standard data extraction form was used to abstract data by two investigators. And the data extraction items included: first author, publication year, region where the study was conducted, ethnicity, case and control definition, allele and genotype frequencies in each group and the method of polymorphism detection. When we encountered an article neither in English nor in Chinese, would a professional translator be consulted to interpret the article.

### Assessment of the risks of bias

Risks of bias were assessed with the following items: 1) Selection bias (cases and controls selections; selections based on disease subtypes), 2) Information bias (genotyping quality control procedures, genotyping under blind conditions and phenotype misclassification rate), 3) Confounding factors (ethnic origin between cases and controls, age and gender distribution between cases and controls).

### Quantitative data synthesis

Hardy-Weinberg equilibrium in the control group of each study was assessed using the chi-square test, while *P* < 0.05 was considered disequilibrium. Paired combinations of genotypes were used to determine the hereditary models: 1) an allelic analysis (L versus S); 2) a genotypic analysis (LL versus SS, LL versus LS, LS versus SS) and 3) another genotypic analysis evaluating dominant or recessive effects of allele L (LL versus LS/SS, LL/LS versus SS). OR and its 95% CI were calculated with the methods recommended by the Cochrane Collaboration [14]. Statistical heterogeneity among studies was detected with the Q test, with a value of *P* < 0.10 indicating heterogeneity existence. The *I*^*2*^ statistics was also employed to assess the risks of heterogeneity: 0%-40% meant no risk of heterogeneity, 30%-60% meant a low risk of heterogeneity, 50%-90% meant substantial heterogeneity and 75%-100% meant considerable heterogeneity [16]. The *I*^*2*^ statistics less than 40% was used as the threshold to determine heterogeneity existence in this meta-analysis. If the Q test and the *I*^*2*^ statistics both indicated no existence of heterogeneity, a fixed model with the Mantel-Haenszel method would be employed to pool data. Otherwise, a random model with the DerSimonian-Laird method would be applied to synthesize data. Funnel plots and Egger’s test were used to examine publication bias [17,18]. The step down Bonferroni method was used for the multiple comparison adjustments [19]. Moreover, Student’s t test and box plots were used to determine allele L variations among different ethnicities. Stata 11.0 software (StataCorp LP, College Station, Texas, USA) was used for meta-analysis, Hardy-Weinberg equilibrium (HWE) tests, Egger’s test, Student’s t test and box plots drawing. R 2.15.0 software (The R Foundation for Statistical Computing, http://cran.rstudio.com/) was used for step down Bonferroni adjustments (Bon). Values of *P* < 0.05 were considered statistically significant for meta-analyses, Bonferroni adjustments, Egger’s test and Student’s t test.

## Results and discussion

### Results

#### Characteristics of selected studies

**Figure 1:**
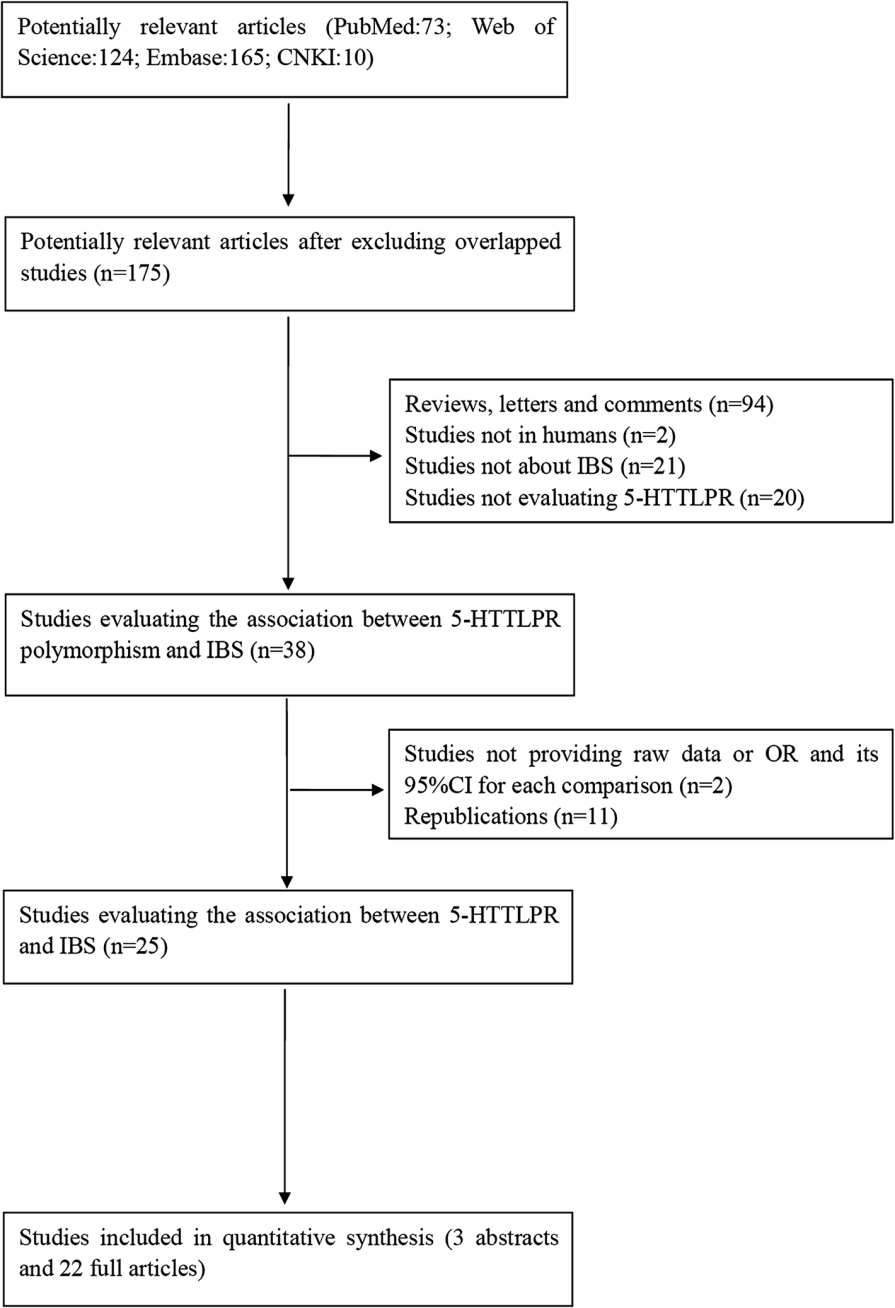
Flow diagram of the study selection process.

A total of 372 potentially relevant publications were identified from the four databases (PubMed: 73; Web of Science: 124; Embase: 165; CNKI: 10). After excluding studies not fulfilling our inclusion criteria including two abstracts not providing genotype frequencies, we included 25 articles (3 abstracts and 22 full text articles) with 3443 IBS cases and 3359 controls into our meta-analysis [10-12,20-41]. Among the selected studies, 11 studies were conducted in the Caucasian population, 9 studies were conducted in the East Asian population, two studies was conducted in the Indian population, one study was conducted in the Iranian population, one study was conducted in the Turkish population and one study was conducted in the Mexican population. In searching reference lists, relevant meta-analyses and reviews, no additional articles were identified. The flow chart of study selection is presented in Figure 1. And the characteristics of selected studies are illustrated in Table 1. The PRISMA Statement is illustrated in Additional file 1. The searching processes of Pubmed, Embase and Web of Science are illustrated in Additional file 2.

**Table 1:** Characteristics of included studies evaluating the association between the 5-HTTLPR polymorphism and the presence of IBS

**Author**	**Year**	**Location**	**Ethnicity**	**Case and control selection**	**SNP method**	**HWE test**
Pata C [20]	2002	Turkey	Turkish	Case: patients diagnosed with Rome I criteria	AS-PCR	
				Control: healthy volunteers without detailed descriptions of matching methods		*P* = 0.0196
Lee DY [21]	2004	Korea	East Asian	Case: patients diagnosed with Rome II criteria	AS-PCR	
				Control: healthy controls without detailed descriptions of matching methods		*P* = 0.8526
Kim HJ [12]	2004	USA	Mostly Caucasian	Case: patients selected from a database of 752 patients with IBS	AS-PCR	
				Control: healthy controls without detailed descriptions of matching methods		*P* = 0.9564
Wang BM [22]	2004	China	East Asian	Case: patients diagnosed with Rome II criteria	AS-PCR	
				Control: healthy contrtols matched for age and gender		*P* = 0.1693
Yeo A [10]	2004	North America	Caucasian	Case: patients diagnosed with Rome I criteria	AS-PCR	
				Control: healthy controls without detailed descriptions of matching methods		*P* = 0.7053
Zhang XM [23]	2006	China	East Asian	Case: patients diagnosed with Rome II criteria	AS-PCR	
				Control: healthy controls matched for age and gender		*P* = 0.9536
Park JM [11]	2006	Korea	East Asian	Case: patients diagnosed with Rome II criteria	AS-PCR	
				Control: healthy controls without detailed descriptions of matching methods		*P* = 0.1976
Whitehead WE [24]	2006	USA	Mostly Caucasian	Case: patients diagnosed with Rome II criteria	AS-PCR	
				Control: healthy controls without detailed descriptions of matching methods		*P =* 0.2376
Li Y [25]	2007	China	East Asian	Case: patients diagnosed with Rome II criteria	AS-PCR	
				Control: healthy controls without detailed descriptions of matching methods		*P* = 0.5862
Saito YA [26]	2007	USA	Mostly Caucasia	Case: patients diagnosed with Rome II criteria	AS-PCR	
				Control: no IBS patients matched for age, race and gender		*P* = 0.2372
Camilleri M [27]	2008	USA	Mostly Caucasian	Case: patients diagnosed with Rome II criteria	AS-PCR	
				Control: healthy controls without detailed descriptions of matching methods		NC
Kohen R [28]	2009	USA	Mostly Caucasia	Case: patients diagnosed with Rome II criteria	AS-PCR	
				Control: healthy volunteers matched for age, race and gender		*P* = 0.1479
Schmulson M [29]	2009	Mexico	Mexican	Case: patients diagnosed with Rome II criteria	AS-PCR	
				Control: healthy controls without detailed descriptions of matching methods		*P* = 0.0000
Niesler B [30]	2010	UK	Caucasian	Case: patients diagnosed with Rome II criteria	AS-PCR	
				Control: healthy volunteers without detailed descriptions of matching methods		*P* = 0.5492
Sikander A [31]	2009	India	Indian	Case: patients diagnosed with Rome II criteria	AS-PCR	
				Control: healthy controls without detailed description of matching methods		*P* = 0.4341
Markoutsaki T [32]	2011	Greek	Caucasian	Case: patients diagnosed with Rome III criteria	PCR-RFLP	
				Control: healthy volunteers without detailed description of matching methods		*P* = 0.6973
Jiang ZD [33]	2012	USA	Mostly Caucasian	Case: patients diagnosed with Rome II criteria	AS-PCR	
				Control: healthy controls without detailed descriptions of matching methods		*P* = 0.1063
Wang YM [34]	2012	China	East Asian	Case: patients diagnosed with Rome III criteria	AS-PCR	
				Control: healthy controls without detailed description of matching methods		*P* = 0.0000
Park CS [35]	2012	Korea	East Asian	Case: patients diagnosed with Rome III criteria	AS-PCR	
				Control: healthy controls without detailed descriptions of matching methods		*P* = 0.3054
Zhang ZX [36]	2012	China	East Asian	Case: patients diagnosed with Rome III criteria	AS-PCR	
				Control: healthy volunteers matched for age, race and gender		*P* = 0.4531
Kumar S [37]	2012	India	Indian	Case: patients diagnosed with Rome III criteria	AS-PCR	
				Control: healthy controls without detailed descriptions of matching methods		*P* = 0.3093
Saito YA [38]	2012	USA	Mostly Caucasia	Case: patients diagnosed with Rome III criteria	AS-PCR	
				Control: healthy examinees matched for age, race and gender		*P* = 0.5924
Colucci R [39]	2013	Italy	Caucasian	Case: patients diagnosed with Rome III criteria	AS-PCR	
				Control: healthy controls without detailed descriptions of matching methods		*P* = 0.6459
Shiotani A [40]	2013	Japan	East Asian	Case: patients diagnosed with Rome III criteria	AS-PCR	
				Control: healthy controls without detailed descriptions of matching methods		*P* = 0.5780
Farjadian S [41]	2013	Iran	Iranian	Case: patients diagnosed with Rome III criteria	AS-PCR	
				Control: healthy examinees matched for age, race and gender		*P* = 0.2284

SNP: single nucleotide polymorphism; PCR-RFLP: polymerase chain reaction-restriction fragment length polymorphism; AS-PCR: allele-specific polymerase chain reaction; HWE test: Hardy-Weinberg equilibrium test; NC: not able to calculated from raw data.

#### Assessment of the risks of bias

##### Selection bias

IBS cases were diagnosed and selected in accordance with the Rome criteria. Because of the evolution of the Rome criteria, all of the three Rome criteria were used by studies included in our meta-analysis. Two studies used Rome I criteria to diagnose IBS [10,20], thirteen studies used Rome II criteria to diagnose IBS [11,21-31,33], nine studies used Rome III criteria to diagnose IBS [32,34-41] and one study selected patients from an IBS database [14]. Only four studies provided detailed descriptions of matching methods [22,23,26,28]. Measures were used to exclude IBS in controls in only seven studies [12,22,26,27,31,35,38]. Most studies selected IBS patients based on IBS subtypes [10-12,20-31,34-41].

##### Information bias

Twenty four studies used allele-specific polymerase chain reaction (AS-PCR) to determine genotypes [10-12,20-31,33-41]. One study used polymerase chain reaction-restriction fragment length polymorphism (PCR-RFLP) to detect genotypes [32]. Two researches were carried out under blind conditions [30,39]. Four studies used the DNA sequencing method to confirm genotypes [11,12,34,41]. All experiments were repeated to ensure consistency for quality control purposes in one study [25]. No phenotype misclassification was reported in the selected studies.

##### Confounding factors

Age and gender distributions were comparable among arms in nine studies [11,22,23,26,28,31,34,35,37], while the other studies did not describe the age and gender distributions among groups. All studies matched cases and controls ethnically.

#### Meta-analysis of the association between 5-HTTLPR polymorphism and IBS

**Figure 2:**
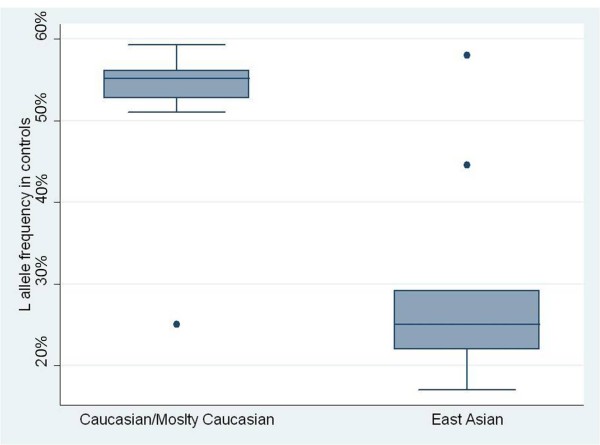
Frequency of the L allele between the East Asian population and the Caucasian population.

Detailed meta-analysis results, models used in each comparison and *P* values for Bonferroni adjustment are shown in Table 2 and Table 3. A wide variation of allele L frequency between the East Asian population and the Caucasian population was found. L allele frequency of the East Asian population controls (29.627%, 95% CI = 19.532%-39.721%) was significantly lower than that of the Caucasian population controls (52.182%, 95% CI = 45.166%-59.198%) (*P* = 0.0003). The box plots are illustrated in Figure 2.

**Table 2:** Meta-analyses of the association between the 5-HTTLPR polymorphism and the presence of IBS

	**Ethnicity**	**Cases/Controls**	**L vs S**			**LL vs SS**			**LL vs LS**		
			**(OR,95% CI)**	***P *(Model)**	**Bon**	**(OR,95% CI)**	***P *(Model)**	**Bon**	**(OR,95% CI)**	***P *(Model)**	**Bon**
IBS											
25 studies	Overall	3443/3359	(0.993,0.867-1.138)	*P* = 0.923 (R)	1.000	(1.009,0.772-1.319)	*P* = 0.948 (R)	1.000	**(1.284,1.040-1.585)**	***P*** **= 0.020 (R)**	0.120
11 studies	Cau/M Cau	1855/1590	(1.008,0.859-1.183)	*P* = 0.919 (R)	1.000	(1.018,0.752-1.378)	*P* = 0.907 (R)	1.000	**(1.288,1.079-1.536)**	***P*** **= 0.005 (F)**	**0.030**
9 studies	East Asian	1138/1102	(1.151,0.901-1.471)	*P* = 0.261 (R)	1.000	(1.371,0.764-2.461)	*P* = 0.291 (R)	1.000	(1.528,0.849-2.751)	*P* = 0.157 (R)	0.942
1 study	Turkish	54/92	(0.934,0.577-1.514)	*P* = 0.782	1.000	(0.727,0.271-1.952)	*P* = 0.527	1.000	(0.442,0.171-1.143)	*P* = 0.092	0.552
2 studies	Indian	301/352	(0.687,0.375-1.259)	*P* = 0.225 (R)	1.000	(0.588,0.241-1.435)	*P* = 0.243 (R)	1.000	(1.077,0.669-1.733)	*P* = 0.761 (F)	1.000
1 study	Mexian	45/123	**(0.482,0.256-0.910)**	***P*** **= 0.024**	0.144	(0.290,0.082-1.030)	*P* = 0.056	0.336	(0.313,0.073-1.337)	*P* = 0.117	0.702
1 study	Iranian	50/100	(1.083,0.670-1.751)	*P* = 0.744	1.000	(1.168,0.466-2.931)	*P* = 0.740	1.000	(2.246,0.976-5.165)	*P* = 0.057	1.000
IBS-C											
20 studies	Overall	992/2437	**(1.232,1.048-1.449)**	***P*** **= 0.011 (R)**	0.066	**(1.570,1.147-2.148)**	***P*** **= 0.005 (F)**	**0.030**	**(1.658,1.180-2.331)**	***P*** **= 0.004 (R)**	**0.024**
8 studies	Cau/M Cau	462/841	**(1.270,1.060-1.520)**	***P*** **= 0.009 (F)**	0.054	**(1.627,1.109-2.388)**	***P*** **= 0.013 (F)**	0.078	(1.269,0.956-1.685)	*P* = 0.100 (F)	1.000
8 studies	East Asian	393/1052	**(1.487,1.139-1.941)**	***P*** **= 0.003 (R)**	**0.018**	**(2.575,1.741-3.808)**	***P*** **= 0.000 (F)**	**0.000**	**(3.084,2.017-4.715)**	***P*** **= 0.000 (F)**	**0.000**
1 study	Turkish	26/92	(0.719,0.379-1.367)	*P* = 0.315	1.000	(0.545,0.156-1.903)	*P* = 0.342	1.000	(0.618,0.172-2.218)	*P* = 0.461	1.000
2 studies	Indian	96/352	(0.798,0.438-1.452)	*P* = 0.460 (R)	1.000	(0.676,0.330-1.384)	*P* = 0.284 (F)	1.000	(0.769,0.389-1.522)	*P* = 0.451 (F)	1.000
1 study	Iranian	15/100	(0.796,0.367-1.725)	*P* = 0.563	1.000	(0.730,0.181-2.951)	*P* = 0.659	1.000	(2.133,0.522-8.714)	*P* = 0.291	1.000
IBS-D											
21 studies	Overall	1454/2813	(0.932,0.771-1.126)	*P* = 0.466 (R)	1.000	(0.888,0.666-1.183)	*P* = 0.416 (R)	1.000	(1.209,0.973-1.502)	*P* = 0.086 (R)	0.516
9 studies	Cau/M Cau	806/1270	(0.986,0.863-1.126)	*P* = 0.835 (F)	1.000	(0.921,0.706-1.200)	*P* = 0.540 (F)	1.000	**(1.251,1.005-1.556)**	***P*** **= 0.045 (F)**	0.270
8 studies	East Asian	434/999	(0.846,0.694-1.032)	*P* = 0.099 (F)	0.594	(0.785,0.498-1.239)	*P* = 0.299 (F)	1.000	(0.930,0.579-1.494)	*P* = 0.764 (F)	1.000
1 study	Turkish	18/92	(1.087,0.529-2.232)	*P* = 0.820	1.000	(0.324,0.015-7.070)	*P* = 0.474	1.000	**(0.046,0.003-0.814)**	***P*** **= 0.036**	0.216
2 studies	Indian	171/352	(0.478,0.084-2.707)	*P* = 0.404 (R)	0.828	(0.494,0.069-3.514)	*P* = 0.481 (R)	1.000	(1.355,0.715-2.566)	*P* = 0.351 (F)	1.000
1 study	Iranian	25/100	(1.325,0.710-2.471)	*P* = 0.377	1.000	(1.565,0.504-4.856)	*P* = 0.439	1.000	**(3.333,1.159-9.586)**	***P*** **= 0.025**	0.150
IBS-M											
17 studies	Overall	486/2042	(1.087,0.911-1.296)	*P* = 0.354 (R)	1.000	(1.170,0.830-1.651)	*P* = 0.370 (R)	1.000	(1.313,0.991-1.740)	*P* = 0.058 (F)	0.348
6 studies	Cau/M Cau	265/549	(1.096,0.754-1.593)	*P* = 0.630 (R)	1.000	(1.153,0.538-2.471)	*P* = 0.714 (R)	1.000	**(1.533,1.069-2.198)**	***P*** **= 0.020 (F)**	0.120
7 studies	East Asian	167/949	(1.030,0.785-1.351)	*P* = 0.830 (F)	1.000	(1.135,0.637-2.022)	*P* = 0.667 (F)	1.000	(0.906,0.507-1.617)	*P* = 0.737 (F)	1.000
1 study	Turkish	10/92	(2.038,0.795-5.225)	*P* = 0.138	0.828	(4.909,0.480-50.178)	*P* = 0.180	1.000	(0.773,0.175-3.415	*P* = 0.734	1.000
2 studies	Indian	34/352	(1.022,0.616-1.697)	*P* = 0.932 (F)	1.000	(1.157,0.452-2.965)	*P* = 0.761 (F)	1.000	(1.684,0.662-4.280	*P* = 0.274 (F)	1.000
1 study	Iranian	10/100	(1.041,0.415-2.610)	*P* = 0.932	1.000	(1.095,0.141-8.485)	*P* = 0.931	1.000	(0.889,0.166-4.755	*P* = 0.891	1.000

Cau/M Cau: Caucasian/Mostly Caucasian; F: fixed model; R: random model; Bon: *P* for Bonferroni adjustment. The significant contrasts were written in bold.

**Table 3:** Meta-analyses of the association between the 5-HTTLPR polymorphism and the presence of IBS

	**Ethnicity**	**Cases/Controls**	**LS vs SS**			**LL vs LS/SS**			**LL/LS vs SS**		
			**(OR,95% CI)**	***P *(Model)**	**Bon**	**(OR,95% CI)**	***P *(Model)**	**Bon**	**(OR,95% CI)**	***P *(Model)**	**Bon**
IBS											
25 studies	Overall	3443/3359	**(0.814,0.681-0.973)**	***P*** **= 0.023 (R)**	0.138	(1.141,0.935-1.391)	*P* = 0.195 (R)	1.000	(0.883,0.739-1.055)	*P* = 0.171 (R)	1.000
11 studies	Cau/M Cau	1855/1590	(0.796,0.566-1.119)	*P* = 0.189 (R)	1.000	(1.506,1.112-2.039)	*P* = 0.070 (F)	0.420	(0.874,0.643-1.189)	*P* = 0.391 (R)	1.000
9 studies	East Asian	1138/1102	(0.892,0.727-1.094)	*P* = 0.272 (R)	1.000	(1.456,0.831-2.549)	*P* = 0.189 (R)	1.000	(0.999,0.828-1.207)	*P* = 0.995 (F)	1.000
1 study	Turkish	54/92	(1.647,0.774-3.505)	*P* = 0.195	1.000	(0.553,0.227-1.348)	*P* = 0.193	1.000	(1.286,0.636-2.599)	*P* = 0.484	1.000
2 studies	Indian	301/352	(0.542,0.284-1.034)	*P* = 0.063 (R)	0.378	(0.763,0.494-1.178)	*P* = 0.223 (F)	1.000	(0.555,0.272-1.130)	*P* = 0.105 (R)	1.000
1 study	Mexian	45/123	(0.929,0.373-2.316)	*P* = 0.875	1.000	(0.295,0.084-.032)	*P* = 0.056	0.336	(0.581,0.268-1.259)	*P* = 0.169	1.000
1 study	Iranian	50/100	(0.520,0.226-1.197)	*P* = 0.124	0.744	(1.770,0.824-3.803)	*P* = 0.143	0.858	(0.697,0.325-1.495)	*P* = 0.354	1.000
IBS-C											
20 studies	Overall	992/2437	(0.926,0.708-1.212)	*P* = 0.578 (R)	1.000	**(1.545,1.187-2.012)**	***P*** **= 0.001 (R)**	**0.006**	(1.119,0.890-1.406)	*P* = 0.335 (R)	1.000
8 studies	Cau/M Cau	462/841	(1.266,0.883-1.815)	*P* = 0.199 (F)	1.000	**(1.327,1.027-1.715)**	***P*** **= 0.031 (F)**	0.186	(1.398,0.994-1.968)	*P* = 0.054 (F)	0.324
8 studies	East Asian	393/1052	(0.906,0.672-1.220)	*P* = 0.514 (F)	1.000	**(2.759,1.933-3.938)**	***P*** **= 0.000 (F)**	**0.000**	(1.250,0.964-1.622)	*P* = 0.093 (F)	0.558
1 study	Turkish	26/92	(0.882,0.337-2.307)	*P* = 0.799	1.000	(0.579,0.180-1.860)	*P* = 0.358	1.000	(0.750,0.312-1.803)	*P* = 0.165	0.990
2 studies	Indian	96/352	(0.783,0.185-3.324)	*P* = 0.740 (R)	1.000	(0.726,0.378-1.394)	*P* = 0.336 (F)	1.000	(0.753,0.213-2.661)	*P* = 0.660 (R)	1.000
1 study	Iranian	15/100	(0.342,0.095-1.234)	*P* = 0.101	0.606	(1.368,0.395-4.734)	*P* = 0.621	1.000	(0.448,0.144-1.391)	*P* = 0.165	0.990
IBS-D											
21 studies	Overall	1454/2813	(0.863,0.619-1.205)	*P* = 0.387 (R)	1.000	(1.042,0.862-1.260)	*P* = 0.668 (R)	1.000	(0.885,0.648-1.209)	*P* = 0.443 (R)	1.000
9 studies	Cau/M Cau	806/1270	(0.854,0.548-1.332)	*P* = 0.423 (R)	1.000	(1.113,0.914-1.357)	*P* = 0.286 (F)	1.000	(0.922,0.626-1.357)	*P* = 0.680 (R)	1.000
8 studies	East Asian	434/999	(0.896,0.686-1.171)	*P* = 0.423 (F)	1.000	(0.850,0.561-1.289)	*P* = 0.444 (F)	1.000	(0.843,0.656-1.083)	*P* = 0.181 (F)	1.000
1 study	Turkish	18/92	**(8.471,1.811-39.626)**	***P*** **= 0.007**	**0.042**	(0.085,0.005-1.462)	*P =* 0.089	0.534	**(5.143,1.115-23.714)**	***P*** **= 0.036**	0.096
2 studies	Indian	171/352	(0.321,0.024-4.370)	*P* = 0.394 (R)	1.000	(0.680,0.214-2.159)	*P =* 0.513 (F)	1.000	(0.371,0.036-3.819)	*P* = 0.405 (R)	1.000
1 study	Iranian	25/100	(0.469,0.152-1.445)	*P* = 0.187	1.000	(2.508,0.986-6.380)	*P =* 0.054	0.324	(0.768,0.286-2.066)	*P* = 0.601	1.000
IBS-M											
17 studies	Overall	486/2042	(0.920,0.706-1.199)	*P* = 0.538 (F)	1.000	(1.186,0.887-1.584)	*P* = 0.250 (R)	1.000	(0.971,0.760-1.242)	*P* = 0.817 (F)	1.000
6 studies	Cau/M Cau	265/549	(0.728,0.476-1.112)	*P* = 0.142 (F)	0.852	(1.172,0.707-1.942)	*P* = 0.539 (R)	1.000	(0.834,0.563-1.237)	*P* = 0.368 (F)	1.000
7 studies	East Asian	167/949	(1.106,0.750-1.632)	*P* = 0.611 (F)	1.000	(1.000,0.590-1.696)	*P* = 1.000 (F)	1.000	(1.082,0.754-1.554)	*P* = 0.669 (F)	1.000
1 study	Turkish	10/92	(6.353,0.727-55.545)	*P* = 0.095	0.570	(1.364,0.325-5.726)	*P* = 0.672	1.000	(5.786,0.703-47.626)	*P* = 0.103	0.618
2 studies	Indian	34/352	(0.695,0.309-1.562)	*P* = 0.379 (F)	1.000	(1.421,0.611-3.303)	*P* = 0.415 (F)	1.000	(0.817,0.393-1.698)	*P* = 0.588	1.000
1 study	Iranian	10/100	(1.232,0.231-6.560)	*P* = 0.807	1.000	(0.940,0.186-4.764)	*P* = 0.941	1.000	(1.195,0.237-6.025)	*P* = 0.829	1.000

Cau/M Cau: Caucasian/Mostly Caucasian; F: fixed model; R: random model; Bon: P for Bonferroni adjustment. The significant contrasts were written in bold.

#### Meta-analysis about IBS

No significant association was found between this polymorphism and IBS in the overall population. In the analysis of the Caucasian population, only the LL vs LS contrast showed a positive result (OR = 1.288, 95% CI = 1.079-1.536, *P* = 0.005, Bon = 0.030). There was no association between the 5-HTTLPR polymorphism and IBS in the East Asian, Iranian, Turkish, Indian and Mexican population.

#### Meta-analysis about IBS-C

**Figure 3:**
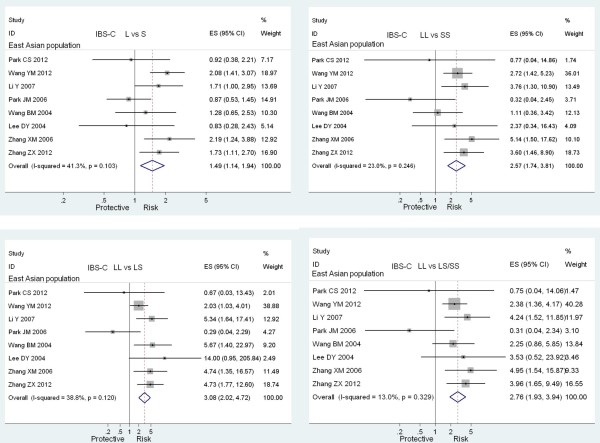
Meta-analyses with positive results of the association between the 5-HTTLPR polymorphism and the presence of IBS-C in the East Asian population.

The LL genotype was a risk factor for IBS-C development in the overall population (LL vs SS: OR = 1.570, 95% CI = 1.147-2.148, *P* = 0.005, Bon = 0.030; LL vs LS: OR = 1.658, 95% CI = 1.180-2.331, *P* = 0.004, Bon = 0.024; LL vs LS/SS: OR = 1.545, 95% CI = 1.187-2.012, *P* = 0.001, Bon = 0.006). In the subgroup analysis of the East Asian population, L allele and LL genotype were significantly associated with increased IBS-C risk in a recessive way (L vs S: OR = 1.487, 95% CI = 1.139-1.941, *P* = 0.003, Bon = 0.018; LL vs SS: OR = 2.575, 95% CI = 1.741-3.808, *P* = 0.000, Bon = 0.000; LL vs LS: OR = 3.084, 95% CI = 2.017-4.715, *P* = 0.000, Bon = 0.000; LL vs LS/SS: OR = 2.759, 95% CI = 1.933-3.938, *P* = 0.000, Bon = 0.000). Forest plots with positive results of the East Asian population are shown in Figure 3. However, there was no significant association between this polymorphism and IBS-C development in the Caucasian, Iranian, Turkish and Indian population. The study conducted in the Mexican population did not evaluate the effect of the 5-HTTLPR polymorphism on IBS-C subtype.

#### Meta-analysis about IBS-D and IBS-M

No significant association was found between this polymorphism and IBS-D and IBS-M in the overall population. In subgroup analysis, there was no significant association between this polymorphism and IBS-C and IBS-M development in the Caucasian, East Asian, Indian and Iranian population. In the analysis between the 5-HTTLPR polymorphism and IBS-D in the Turkish population, only the LS vs SS contrast showed a positive result (OR = 8.471, 95% = 1.811-39.626, *P* = 0.007, Bon = 0.042). No association was found between this polymorphism and IBS-M development in the Turkish population. The study conducted in the Mexican population did not evaluate the effect of this polymorphism on IBS-D and IBS-M subtypes.

#### Sensitivity analysis

After excluding studies not fulfilling HWE or not providing HWE data, the conclusions of our meta-analysis were not changed. The conclusions were not changed either when we used both a fixed and a random model to perform meta-analyses.

#### Evaluation of publication bias

**Figure 4:**
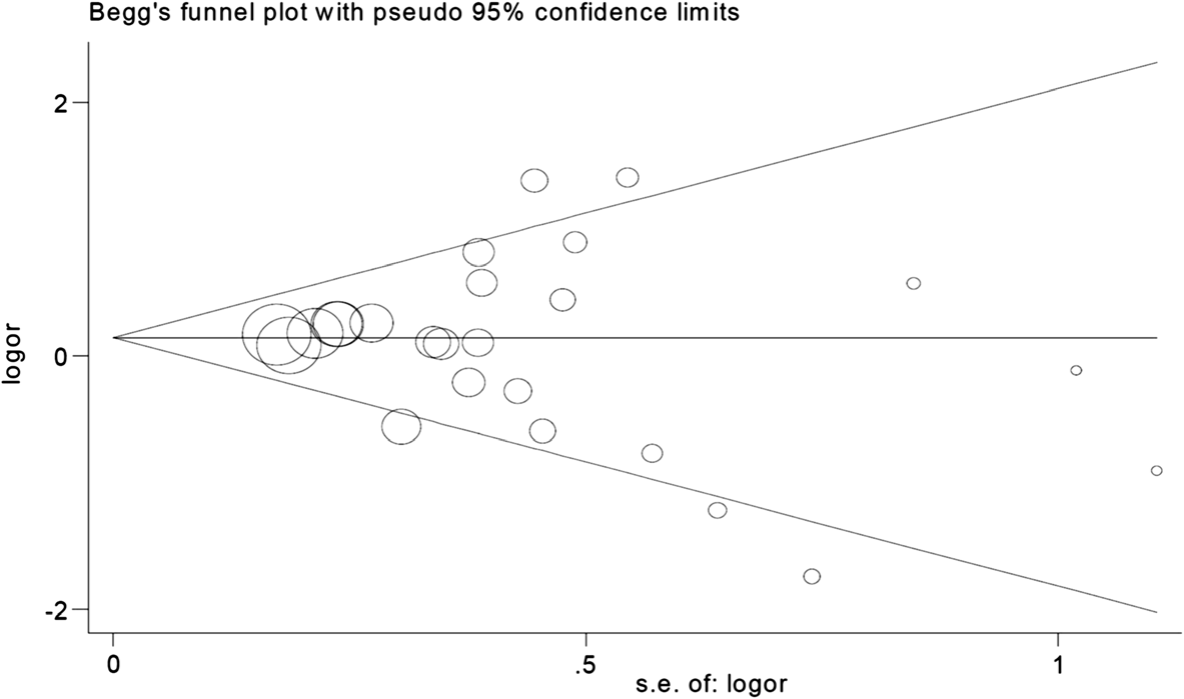
Begg funnel plot of the overall IBS analysis LL vs LS/SS.

The Begg funnel plot was symmetry in the overall IBS analysis, as shown in Figure 4. Begger’s test showed no publication bias in the overall IBS analysis (*P* = 0.529).

## Discussion

Gut motility dysfunction, visceral hypersensitivity and psychopathological factors have been implicated to play key roles in the development of IBS [1,3]. Recently, some scholars integrated the above factors and postulated a model called brain-gut axis (BGA), which is bi-directional and constitutes the enteric nervous system (ENS) and the gut wall, CNS and the hypothalamus-pituitary-adrenal (HPA) axis [42]. BGA dysfunction at each level is believed to be involved in the pathophysiology of IBS [43]. Psychopathological factors cause gut motility dysfunction, abnormal mucosa secretion and visceral hypersensitivity via BGA and vice versa. So BGA model may be the optimal model in understanding IBS pathophysiology and can explain most of the presentations of IBS frequently accompanied with psychological conditions.

Neural, endocrine and neuro-immune pathways are all involved in BGA. And neurotransmitters are involved in all the three pathways, so inappropriate neurotransmitter secretion could cause BGA dysfunction and plays a key role in IBS development. Serotonin existing in not only GI tracts but also CNS is one of these neurotransmitters. Changes of the serotonin levels in GI tracts can affect visceral sensitivity and gut motility [7,44]. Previous studies have also showed that elevated plasma serotonin is associated with IBS-D and decreased plasma serotonin is associated with IBS-C [7]. Once serotonin is secreted from EC cells, SERT will be activated to reuptake serotonin back into EC cells and attenuate the effect of serotonin in GI tracts subsequently [8]. The L variation of 5-HTTLPR is found to increase the expression of SERT gene and enhance SERT activity consequently [9-11,34]. The enhanced SERT activity would take up serotonin and weaken its effects in promoting gut secretion and motility. So compared with S allele and SS genotype carriers, L allele and LL genotype carriers are likely to be more susceptible to IBS-C. The results of our meta-analysis support this hypothesis. However, the effect of 5-HTTLPR on IBS-C is population dependent and the positive association is only present in the East Asian population but not in the Caucasian population. We also found that L allele frequency of the East Asian population controls was significantly lower than that of the Caucasian population controls. So the different allelic frequency between the two populations may account for this phenomenon. Furthermore, there is a higher prevalence of psychological and psychiatric disorders among IBS patients [45]. The state of CNS can also affect the development of IBS. However, the effect of the 5-HTTLPR variant on CNS is different from that on GI tracts in IBS development. A meta-analysis demonstrated that the S variant was associated with heightened amygdala activation which would predispose S carriers to stress-related psychiatric disorders [46]. Another meta-analysis also showed a positive association between the S allele and an increased risk of developing depression under stress [47]. The S allele may increase the risk of IBS-C development through the CNS pathway. So the opposite effect of 5-HTTLPR variant on GI tracts and CNS during IBS-C development may also account for some variations among studies assessing the association between the 5-HTTLPR variant and IBS-C. Future studies should exclude the psychological confounding factors or stratify analyses based on psychological conditions. IBS may be further categorized into subtypes based on psychological conditions besides of the Rome criteria. Additionally, an adenine to guanine polymorphism in the L allele has been recently identified and only the adenine L allele but not the guanine L allele is found to increase the activity of SERT [48]. This internal L allele polymorphism may attenuate the effect of the L allele on IBS-C development. Only one small sample size study included in our meta-analysis evaluated the effect of internal L allele polymorphism on IBS development and showed a negative result. Future studies should further assess the effect of the 5-HTTLPR polymorphism on IBS-C according to the internal L allele polymorphism. Moreover, a study indicates that the S allele is associated with higher pain sensory ratings during rectal distension studies in healthy controls and IBS patients, and the increased sensation ratings in carriers of the S allele are not caused by lower rectal compliance [27]. Different genotype and allele may have different effects on gut motility and intestinal sensation respectively. This phenomenon also indicates the complexity of IBS and the necessity to classifying IBS based on pathophysiology.

Clarifying genotypes of the 5-HTTLPR polymorphism also has clinical implications. One study found that IBS-C patients with the LL genotype responded poorly to treatment with the 5-HT4 receptor agonist, tegaserod [25]. Another study investigating rectal smooth muscle contractions found that IBS patients with the SS genotype showed more increase in phasic contractions compared with patients with the LL genotype after administration of the 5-HT4 receptor agonist, mosapride [49]. Determining the genotype of the 5-HTTLPR polymorphism may be of value to the prognosis and the prediction of treatment response in a IBS-C patient.

The conclusions of this meta-analysis are a little different from the previous meta-analyses. As we know, this meta-analysis is the latest one and has the largest sample size. So the result of our meta-analysis is more likely convincing. However, the conclusions of this meta-analysis should be interpreted cautiously due to some limitations. Firstly, selection bias could not be excluded as only seven studies employed measures to exclude IBS in the controls. Secondly, only four studies used the DNA sequencing method to confirm genotypes, and two studies used blindness measures. Information bias is inevitable. Thirdly, most of the included studies did not describe the age and gender distribution between cases and controls. Confounding factors could not be excluded. Moreover, heterogeneity existed in some contrasts which might also affect the validity of this meta-analysis. Although the Egger’s test showed no publication bias existing, the data of two abstracts were not included in this meta-analysis for failing to provide genotype frequencies. Thus a reporting bias existed. Moreover, we employed the Bonferroni adjustments method to avoid false positive results, the risk of false negative results occurrence would be increased. So when interpreting the negative results of our meta-analysis, we should be cautious.

## Conclusions

In summary, in despite of the above limitations, this meta-analysis shows a positive association between the L allele and LL genotype of 5-HTTLPR mutation and IBS-C in the East Asian population but not in the Caucasian population. These results offer some insights into gene functions affecting IBS susceptibility and some clues in IBS management, especially in the East Asian population.

## Competing interests

The authors declare that they have no competing interests.

## Authors’ contributions

Conceived and designed the experiments: ZFZ and ZJD. Performed the experiments: ZFZ, LXW, DY, GZ, LZ. Searched, selected, analyzed the data: ZFZ, LXW, DY, GZ, LZ. Wrote the paper: ZFZ, LXW, DY. All authors read and approved the final manuscript.

## Pre-publication history

The pre-publication history for this paper can be accessed here:

http://www.biomedcentral.com/1471-230X/14/23/prepub

## Supplementary Material

Additional file 1 The PRISMA Statement. Click here for file

Additional file 2 The searching process of Pubmed, Embase and Web of Science. Click here for file
