# Supplementary material for: The serotonin transporter gene polymorphism (5-HTTLPR) and irritable bowel syndrome: a meta-analysis of 25 studies
Source: BMC Gastroenterol. 2014 Feb 10;14:23. doi: 10.1186/1471-230X-14-23 (PMC3926682; doi:10.1186/1471-230X-14-23)
Supplement: Additional file 2 — The searching process of Pubmed, Embase and Web of Science. [file 1471-230X-14-23-S2.doc]

| Search | Query | Items found |
| --- | --- | --- |
| #16 | Search ((#13) AND #14) AND #15 | 73 |
| #15 | Search ((((((#6) OR #7) OR #8) OR #9) OR #10) OR #11) OR #12 | 571618 |
| #14 | Search ((#3) OR #4) OR #5 | 126984 |
| #13 | Search (#1) OR #2 | 9455 |
| #12 | Search polymorphism | 214803 |
| #11 | Search polymorphisms | 216318 |
| #10 | Search single nucleotide | 130080 |
| #9 | Search allele | 180760 |
| #8 | Search alleles | 125749 |
| #7 | Search genotypes | 328735 |
| #6 | Search genotype | 310008 |
| #5 | Search 5-HT | 124899 |
| #4 | Search 5-Hydroxytryptamine | 124718 |
| #3 | Search serotonin | 121659 |
| #2 | Search IBS | 4960 |
| #1 | Search irritable bowel syndrome | 8256 |

Pubmed (2013-7-12)

Web of science (2013-712)

| ID | Query | Result |
| --- | --- | --- |
| #16 | #13 AND #14 AND #15 | [124](http://apps.webofknowledge.com.eproxy.ucd.ie/summary.do?product=WOS&doc=1&qid=33&SID=Z1F651NHdLA7p3JeCPk&search_mode=CombineSearches) |
| #15 | #6 OR #7 OR #8 OR #9 OR #10 OR #11 OR #12 | [512,142](http://apps.webofknowledge.com.eproxy.ucd.ie/summary.do?product=WOS&doc=1&qid=32&SID=Z1F651NHdLA7p3JeCPk&search_mode=CombineSearches) |
| #14 | #3 OR #4 OR #5 | [102,427](http://apps.webofknowledge.com.eproxy.ucd.ie/summary.do?product=WOS&doc=1&qid=24&SID=Z1F651NHdLA7p3JeCPk&search_mode=CombineSearches) |
| #13 | #1 OR #2 | [14,502](http://apps.webofknowledge.com.eproxy.ucd.ie/summary.do?product=WOS&doc=1&qid=20&SID=Z1F651NHdLA7p3JeCPk&search_mode=CombineSearches) |
| #12 | Topic=(polymorphism) | [263,964](http://apps.webofknowledge.com.eproxy.ucd.ie/summary.do?product=WOS&doc=1&qid=31&SID=Z1F651NHdLA7p3JeCPk&search_mode=GeneralSearch) |
| #11 | Topic=(polymorphisms) | [263,964](http://apps.webofknowledge.com.eproxy.ucd.ie/summary.do?product=WOS&doc=1&qid=31&SID=Z1F651NHdLA7p3JeCPk&search_mode=GeneralSearch) |
| #10 | Topic=(single nucleotide) | [77,625](http://apps.webofknowledge.com.eproxy.ucd.ie/summary.do?product=WOS&doc=1&qid=29&SID=Z1F651NHdLA7p3JeCPk&search_mode=GeneralSearch) |
| #9 | Topic=(allele) | [165,681](http://apps.webofknowledge.com.eproxy.ucd.ie/summary.do?product=WOS&doc=1&qid=27&SID=Z1F651NHdLA7p3JeCPk&search_mode=GeneralSearch) |
| #8 | Topic=(alleles) | [165,681](http://apps.webofknowledge.com.eproxy.ucd.ie/summary.do?product=WOS&doc=1&qid=28&SID=Z1F651NHdLA7p3JeCPk&search_mode=GeneralSearch) |
| #7 | Topic=(genotypes) | [223,913](http://apps.webofknowledge.com.eproxy.ucd.ie/summary.do?product=WOS&doc=1&qid=26&SID=Z1F651NHdLA7p3JeCPk&search_mode=GeneralSearch) |
| #6 | Topic=(genotype) | [223,913](http://apps.webofknowledge.com.eproxy.ucd.ie/summary.do?product=WOS&doc=1&qid=25&SID=Z1F651NHdLA7p3JeCPk&search_mode=GeneralSearch) |
| #5 | Topic=(5-HT) | [25,961](http://apps.webofknowledge.com.eproxy.ucd.ie/summary.do?product=WOS&doc=1&qid=23&SID=Z1F651NHdLA7p3JeCPk&search_mode=GeneralSearch) |
| #4 | Topic=(5-Hydroxytryptamine) | [20,216](http://apps.webofknowledge.com.eproxy.ucd.ie/summary.do?product=WOS&doc=1&qid=22&SID=Z1F651NHdLA7p3JeCPk&search_mode=GeneralSearch) |
| #3 | Topic=(serotonin) | [86,940](http://apps.webofknowledge.com.eproxy.ucd.ie/summary.do?product=WOS&doc=1&qid=21&SID=Z1F651NHdLA7p3JeCPk&search_mode=GeneralSearch) |
| #2 | Topic=(IBS) | [5,991](http://apps.webofknowledge.com.eproxy.ucd.ie/summary.do?product=WOS&doc=1&qid=19&SID=Z1F651NHdLA7p3JeCPk&search_mode=GeneralSearch) |
| #1 | Topic=(irritable bowel syndrome) | 3,871 |

Embase(2013-7-12)

| ID | Query | Result |
| --- | --- | --- |
| #16 | #13 AND #14 AND #15 | [165](http://proxy.library.upenn.edu:2592/search/results?viewsearch=16) |
| #15 | #6 OR #7 OR #8 OR #9 OR #10 OR #11 OR #12 | [794,364](http://proxy.library.upenn.edu:2592/search/results?viewsearch=15) |
| #14 | #3 OR #4 OR #5 | [333,993](http://proxy.library.upenn.edu:2592/search/results?viewsearch=14) |
| #13 | #1 OR #2 | [14,452](http://proxy.library.upenn.edu:2592/search/results?viewsearch=13) |
| #12 | polymorphism | [320,041](http://proxy.library.upenn.edu:2592/search/results?viewsearch=12) |
| #11 | polymorphisms | [110,746](http://proxy.library.upenn.edu:2592/search/results?viewsearch=11) |
| #10 | singleAND ('nucleotide'/exp OR nucleotide) | [310,589](http://proxy.library.upenn.edu:2592/search/results?viewsearch=10) |
| #9 | 'allele'/exp OR allele | [183,165](http://proxy.library.upenn.edu:2592/search/results?viewsearch=9) |
| #8 | 'alleles'/exp OR alleles | [158,804](http://proxy.library.upenn.edu:2592/search/results?viewsearch=8) |
| #7 | genotypes | [85,629](http://proxy.library.upenn.edu:2592/search/results?viewsearch=7) |
| #6 | 'genotype'/exp OR genotype | [287,847](http://proxy.library.upenn.edu:2592/search/results?viewsearch=6) |
| #5 | '5 ht'/exp OR '5 ht' | [111,598](http://proxy.library.upenn.edu:2592/search/results?viewsearch=5) |
| #4 | '5 hydroxytryptamine'/exp OR '5 hydroxytryptamine' | [96,333](http://proxy.library.upenn.edu:2592/search/results?viewsearch=4) |
| #3 | 'serotonin'/exp OR serotonin | [331,136](http://proxy.library.upenn.edu:2592/search/results?viewsearch=3) |
| #2 | ibs | [8,571](http://proxy.library.upenn.edu:2592/search/results?viewsearch=2) |
| #1 | irritableAND ('bowel'/exp OR bowel) AND ('syndrome'/exp OR syndrome) | [11,400](http://proxy.library.upenn.edu:2592/search/results?viewsearch=1) |
